# Effects of household vectors on child health and its determinants in southwest, Ethiopia correspondence analysis

**DOI:** 10.3389/fpubh.2024.1341422

**Published:** 2024-03-06

**Authors:** Bezuayehu Alemayehu, Besufekad Mekonnen, Abebaw Addisu, Abyot Asres

**Affiliations:** ^1^Department of Environmental Health, Mizan Tepi University Ethiopia, Mīzan Teferī, Ethiopia; ^2^Department of Public Health, Mizan Tepi University Ethiopia, Mīzan Teferī, Ethiopia

**Keywords:** child, Correspondence analysis, effects, health, household vectors

## Abstract

**Background:**

Household vectors transmit pathogens from one child to another. This study aimed to investigate the prevalence and determinants of household vectors on child health.

**Methods:**

A community-based cross-sectional study design was conducted, during which a total of 846 household data were collected using a pretested questionnaire and simple random sampling technique. The data was entered into EpiData3.4 and then exported to Stata 14 software for analysis. A multivariable logistic regression analysis was conducted to identify significant factors associated with household vectors that contribute child health problems. The correspondence analysis was used to determine statistically significant effects or associations between household vectors and child health problems, that was explained by the percentage of variance.

**Results:**

This study revealed that the prevalence of household vector effects among children was 35.5% suchas itching, allergies, nuisances and aesthetically displeasing factors. Households with no formal education were significantly 36% less likely to be affected compared to their counterparts (AOR 0.64, 95% CI 0.41, 0.99). Housewives are significantly 2.21 times more likely to be bexposed to household vectors compared to government workers (AOR 2.21, 95% CI 1.23, 3.70). Caregivers who had limited awareness of household vectors were 98.6% less likely to be affected compared to their counterparts (AOR 0.014; 95% CI 0.01–0.04). Similarly, children from households that consumed less than 20 liters of water per individual were 1.45 times more likely to be at risk compared to children from households that consumed more water (AOR 1.45, 95% CI 1.02, 2.07). The infestations of household vectors were found to be statistically significant and were associated with the occurrence of child health problems. This significant association accounted for 86.8 percent of the explained variance.

**Conclusion:**

Addressing the high burden of household vectors on child health requires interventions that target informal education, limited access to information, and inadequate access to safe water. Implementing effective vector control measures is crucial to reduce the incidence of vector-borne diseases among children.

## Introduction

Household vectors are genera that have a history of living in or invading, human habitation causing disease or acting as disease vectors, and posing other threats (1, 2). A disease vector is any living agent that carries and transmits an infectious pathogen such as a parasite or microbe, to another living organism (3). Arthropods form a major group of pathogen vectors with mosquitoes, flies, lice, fleas, ticks, and mites transmitting a huge number of pathogens. Many such vectors are hematophagous, which feed on blood at some or all stages of their lives. When the insects feed on blood, the pathogen enters the bloodstream of the host. This can happen in different ways directly by bites, stings, or infestation of tissues, or indirectly through disease transmission (1, 4).

WHO issued reports indicating that vector-borne infections disproportionately affetct underprivileged individuals, particularly those residing in regions lacking proper sanition, clean drinking water and adequate housing. It is estimated that over 80% of the world’s population resides in areas under threat of at least one vector-borne disease, where there is a “Small bite, big threat” (5, 6). Mainly asthma episodes triggered by exposure to dust mites, cockroaches, pets, and rodents which are more pronounced among children because the children’s internal organs are still developing and maturing.

The previous study conducted in Jimma town revealed that 83.6% of respondents had sufficient knowledge and utilized appropriate methods for vector control. The most commonly practiced method was the application of smoke by burning repellent materials.

Different evidence indicates various effects of cockroaches such as producing odorous secretions and harming food quality. They also carry pathogens on their surfaces which can cause diseases including food poisoning, nausea, vomiting, and diarrhea. Furthermore, cockroaches carry a wide variety of harmful microorganisms inside their bodies and contain allergens, in their excrement and cast-off skins. These allergens can cause rashes, watery eyes and sneezing (7). The link between allergies and cockroaches has been documented in the past 30 years. Exposure to normal secretions of cockroaches such as their feces or shed nymph skin can cause an asthmatic reaction in people. For example in houses infested by cockroaches, sensitivity among asthmatic children is as high as 79% (8), these insects are proven or suspected carriers of disease-causing organisms such as diarrhea, dysentery, cholera, plague, typhoid fever, and viral diseases like poliomyelitis. In addition, they carry the eggs of parasitic worms and may cause allergic reactions, such as dermatitis, itching, swelling of the eyelids and more serious respiratory conditions (9). The problem of household insects is more prevalent among the poor socio-economic community, which lacks awareness about their role in disease transmission.

Epidemic relapsing fever is a louse-borne infection caused by the spirochete Borreliarecurrentis. It affected several million people worldwide during the first half of the 20^th^ century, especially during the world wars Flea-borne infections are emerging or re-emerging in various parts of the world (10). The flea affects many impoverished populations living in sub-Saharan Africa, the Caribbean, and South America. This menace has also resulted in school dropouts and it is estimated that over 2 million people in Kenya require assistance with the jigger problem (11).

Different studies have indicated that knowledge, attitude and practices regarding vector infestation among households show that 70.1% acknowledge that poor hygiene and sanitation contribute to health problems related to jigger infestation among children (11). Unsanitary practices create a breeding ground for diseases, which are often carried from one site to another by insects and easily transmitted among people living in proximity (12). However, there is limited information available and a lack of evidence on the effects of household vectors on child health in southwestern Ethiopia. Therefore, this study aims to identify and address these information gaps. It will examine how the burden of household vectors varies depending on the types of vectors and various contributing factors such as community awareness and socioeconomic status of the community.

## Materials and methods

### Study area

**Figure 1:**
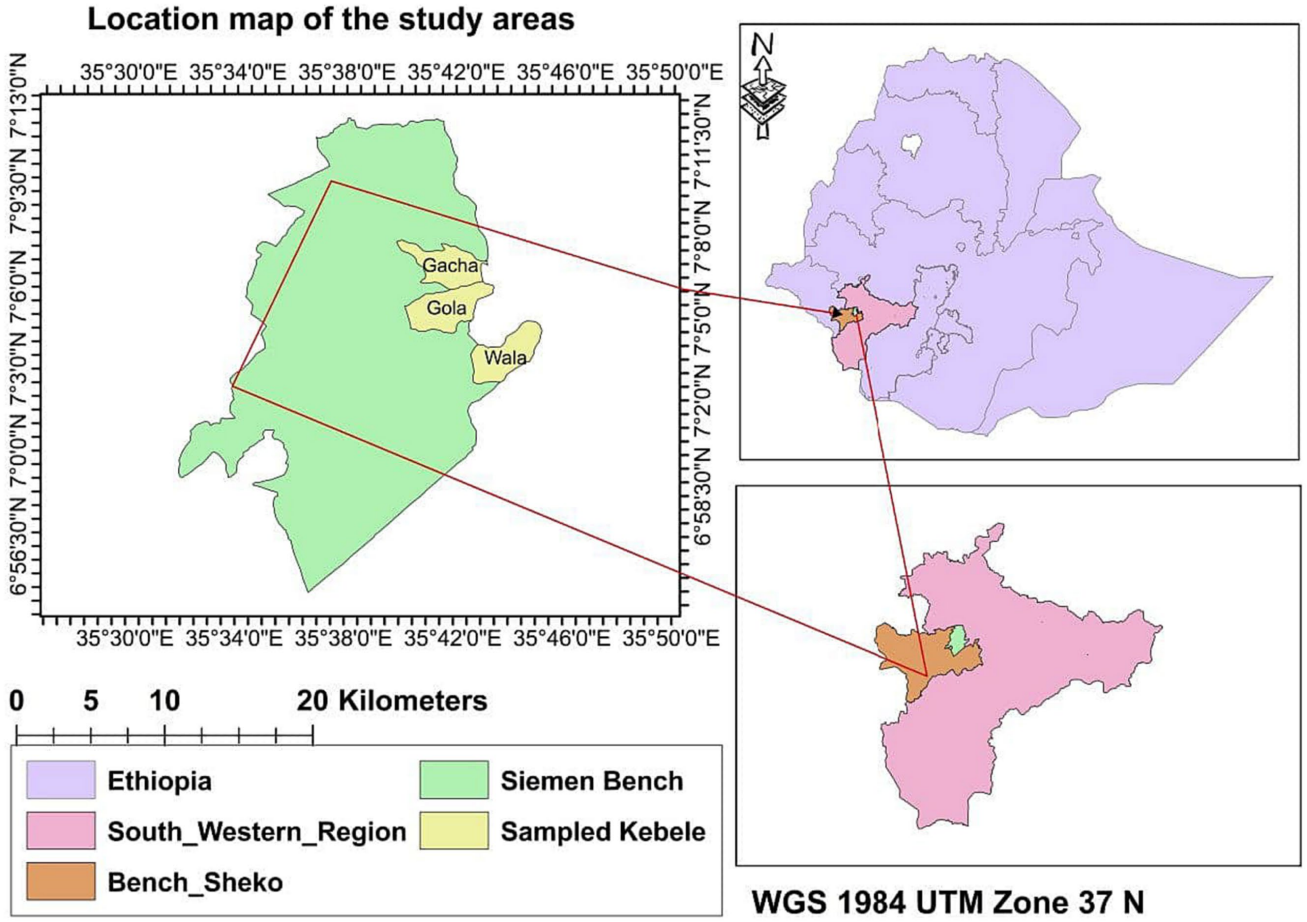
Map of the study area.

The study was conducted in the Semen Bench district, Bench Sheko, southwestern Ethiopia from December 1, 2021, to February 30, 2022. This district is located 550 kilometers away from Addis Ababa, Ethiopia. The total population in the study area was 148,285, of which 71,177 were men, 77,108 were women and 23,147 were children under 5 years old. The study area consists of 24 kebeles with a total of 29,610 households. The average family size in this household is 4.14. The map of the study area was created using ArcGIS 10.5 to show the sampled kebeles including Gacha, Wala, and Gola (Figure 1), These kebeles have a household account of 912,1,200 and 1,350, respectively.

### Study design and period

A community-based cross-sectional study design was used to investigate the burden of household vectors on child health in resource-limited areas of southwestern Ethiopia from December to March 2022.

### Source and study population

The source population consisted of the residents of the Semen Bench district, whereas, the study population on the other hand included households that had been living in their communities for 6 months before data collection.

Mothers who were unable to communicate or non-voluntary commitments were excluded from the study.

### Sample size determination and sampling technique

To conduct this research, the sample size was determined using a single population formula taking into account the following factors:95% confidence interval (CI), a margin of error (d), 50% burden prevalence (p) of household vectors on child health, design effects of 2 and a 10% non-response rate (13). Finally, a total sample size of 846 was obtained to investigate the burden of household vectors on child health in resource-limited areas of southwestern Ethiopia (40). Uneven distributions of sample sizes were utilized and applied to the selected kebeles.

$n=\;\frac{{\left(z\alpha /2\right)}^{2}\left(1-p\right)*0.5}{d^{2}}$ , Where, n = sample size, q = 1-p, d = margin of error = 0.05 (5%).

*p* = 0.5%; the prevalence of households’ knowledge about common household insects has not been studied before. A simple random sampling technique was used to select HHS from selected Kebele using a proportional allocation approach. Especially Gacha, Wala, and Gola kebele were sampled with 223, 293, and 330 households, respectively.

### Inclusion and exclusion criteria

Mothers or households with children who are residing for at least 6 months and above 18 years old were included in this study. Mothers who were unwilling to participate due to certain health problems such as mental issues and those who were not found at home after repeated visits were excluded.

### Data collection process

A structured questionnaire was adopted from similar studies in line with the objectives of this study. The checklist was utilized to observe and record data regarding the presence of household vectors, child health problems, housing conditions, and the presence of domestic animals in the same residence.

### Data quality and pre-test

The pretest was conducted outside of the actual study area with a similar socio-economic setup. After the pretest results a necessary amendment was made. The study participants were informed about the effects of household vectors to address potential sources of recall biases. Data collectors received 2 days of training. The data collection process was conducted under the supervision of the researchers to ensure data quality.

### Operational definition

**Outcome variable:** The burden of household vectors on child health is defined as the health problems experienced by children under the age of five. These problems may include itching, nuisances, signs of allergens, aesthetically displeasing conditions, the spread of diseases, contamination of utensils and food sources.

**Knowledge:** The respondents’ mental process which includes thinking, remembering, perceiving, planning and choosing, is related to their views on common household insects. This is based on the questions provided.

**Attitude:** The feelings and beliefs of respondents regarding common household insects.

**Practice:** The actions intended to prevent common household insects.

### Statistical analysis

The data was processed and entered into EpiData Manager 4.02.101 before being exported to Stata version 14 for analysis. ArcGIS10.5 software was used to create maps of the selected study areas. Frequencies and percentages were used to summarize the variables studied. The variance inflation factor (VIF) a measure of collinearity was used to examine multicollinearity between independent variables. VIF less than ten (11) were considered (14, 15). The chi-square test was used to determine the effects of household vectors on the health-identified child. A binary logistic regression model was utilized for multivariable analysis to identify potential variables associated with the effects of household vectors on child health. Variables with a value of p less than 0.25 were considered significant.

The output of the final model was interpreted using an adjusted odds ratio (AOR) with a 95%confidence interval at a statistical significance threshold of 0.05.

The correspondence analysis was applied to identify or check the statistically significant effects/ association of household vectors with child health problems which could be explained by the percentage of variance under the biplot of the observation. The percentage of variance explained accounts for the different dimensions or the extent of the child’s health problem attributed to household vectors. The first dimension accounts for the most effects, followed by the second dimension, and then the third dimension, etc., which is indicated by the correspondence analysis image (16).

## Results

The sociodemographic characteristics of the respondents are presented in Table 1. A total of 771 households were involved in this study with a response rate of 91.13%. The mean age of the study participants was 34.18 ± 6.75 with 76.5 being married caregivers and approximately 60.2% being females. The education status of caregivers was 47.9% with primary education attendants including 45.9% of farmers as occupational status (Table 2).

**Table 1:** Socio-demographic characteristics of the respondents.

Variables of household		Frequency	Percept (%)
Mean age		34.18 ± 6.75	
Marital status	Single	181	23.5
	Married	590	76.5
Family members	4.09 ± 0.56		
Sex	Male	307	39.8
	Female	464	60.2
Education status of caregivers	Primary	3 69	47.9
	Secondary	272	35.3
	Diploma	130	16.9
Occupational status of caregivers	Housewife	144	18.7
	Daily worker	121	15.7
	Private	69	8.9
	Farmer	354	45.9
	Gov. worker	83	10.8

**Table 2:** Identified household vectors found in the home.

Variables		Category	Frequency	Percept (%)
Effects of household insects		Yes	274	35.5
		No	497	64.5
Identified household vectors found in the home				
House fly		Yes	351	45.5
		No	420	54.5
Cock roaches		Yes	439	56.9
		No	332	43.1
Fleas		Yes	473	61.3
		No	298	38.7
Lice		Yes	487	63.2
		No	284	36.8
Mosquito		Yes	326	42.3
		No	445	57.7
Bedbugs		Yes	507	65.8
		No	264	34.2
Ticks		Yes	501	65
		No	270	35
Knows ways of disease transmission mechanism by vectors	Bites	Yes	89	11.5
		No	682	88.5
	Stings, a deposit of infective material on food	Yes	83	10.8
		No	688	89.2
	Deposit of infective material on the skin	Yes	86	11.2
		No	685	88.8
	Infestation of tissues	Yes	87	11.3
		No	684	88.7
Sources of information for effects of common household vectors				
TV		Yes	89	11.5
		No	682	88.5
Friend		Yes	102	13.2
		No	669	86.8
Health facility		Yes	97	12.6
		No	674	87.4
Health workers		Yes	130	16.9
		No	641	83.1
Health effects of household vectors on child health	Nuisance	Yes	255	33.1
		No	516	66.9
	Itching	Yes	259	33.6
		No	512	66.4
	Contaminate utensils and food sources	Yes	265	34.4
		No	506	65.6
	Spread disease	Yes	271	35.1
		No	500	64.9
	Aesthetically displeasing	Yes	255	33.1
		No	516	66.9
	An allergen source	Yes	263	34.1
		No	508	65.9
Cause of insect infestation	Dirty environment	Yes	273	35.4
		No	498	64.6
	Poor personal hygiene	Yes	270	35
		No	501	65
	Lack of awareness about insects	Yes	271	35.1
		No	500	64.9
	Poor housing condition	Yes	269	34.9
		No	502	65.1
Believe that insects can transmit diseases?		Yes	276	35.8
		No	495	64.2
Feel discomfort due to control of insects when using local modern methods practice?		Yes	282	36.6
		No	489	63.4
HH insects cannot be controlled?		Strongly agree	44	5.7
		Agree	36	4.7
		Strongly disagree	70	9.1
		Disagree	124	16.1
		NA	497	64.5

### Identified household vectors found in the home

Table 2 indicates the identified household insects present in the home. The study found that the overall prevalence of household insect effects among children was 35.5%. The household insects that were Identified as present in the home causing various health effects among the children were house flies 351 (45.5%), cock roaches 439 (56.9%), fleas 473 (61.3%), lice 487 (63.1%), and bedbugs 507 (65.%). Moreover, the respondents are aware of various ways diseases can be transmitted including through bites 89 (11.5%), stings 83 (10.8%), a deposit of infective material on food 86 (11.2%), a deposit of infective material on the skin 86 (11.2%) and infestation of tissues 87 (11.3%).

The sources of information for the study participants regarding the effects of household vectors were television, friends, health facilities and health workers. Additionally, the specific health effects of household vectors on child health were nuisance 255 (33.1%), itching 259 (33.6%), contaminated utensils and food sources 265 (34.4%), spreading disease 271 (35.1%) aesthetically displeasing 255 (33.1%) and an allergen source 263 (34.1%). The cause of insect infestation mentioned was a dirty environment 273 (35.4%), poor personal hygiene 270 (35%), lack of awareness about insects 271 (35.1%), and poor housing conditions 269 (34.9%). The majority of the respondents believe that insects can transmit diseases 276 (36.6%) that can be controlled 124 (16.1%).

### Traditional methods and practices to prevent household vectors

Table 3 indicates the traditional methods and practices to prevent household vectors. Practices such as burning wood/smoking (54.1%), always keeping the child’s hygiene (45.4%), cleaning all rooms (40.1%) and other water, hygiene, and sanitation practices. While modern solutions exist, traditional, methods and practices are still effective in preventing household vectors. by following these tips, creating an environment that is unattractive to insects ensuring vector-free home families.

**Table 3:** Traditional methods and practices to prevent household vectors.

Practices				
Appropriate traditional methods to control measures you took?	Burning wood/smoking	Yes	417	54.1
		No	354	45.9
	Always keeping child’s hygiene	Yes	350	45.4
		No	421	54.6
	Cleaning all rooms	Yes	310	40.1
		No	461	59.9
	Using plants as lotion	Yes	269	34.9
		No	502	64.1
Water, hygiene and sanitation				
Sources of water for HH?	Improved	285	37	
	unimproved	486	63	
	Daily HH water demand in a litter	235	30.5	
		536	69.5	
Liquid waste management practice	Yes	208	26.9	
	No	564	73.1	
Solid waste management practice	Yes	135	17.5	
	No	636	82.5	
Pit latrine facility	Yes	548	71.1	
	No	223	28.9	
Handwashing water with soap	Yes	168	21.8	
	No	603	78.2	
Housing conditions, cleanness, by observation	Yes	274	35.5	
	No	497	64.5	
Households share with domestic animals	Yes	228	29.5	
	No	543	70.4	

### Determinants of the effects of household vectors among children

The results of the multivariable analyses are displayed in Table 4. It was found the prevalence of the effects of households insects in this study area is 35.5%. This indicates that individuals without formal education had a 36.5% lower risk of experiencing effects compared to households with an educational level above secondary education (AOR 0.64, 95% CI 0.41, 0.99). Similarly, the effect among housewives was 21% (AOR 2.21, 95% CI 1.23, 3.70) higher than among government workers a significant effect (value of *p* <0.005) among students, private workers and farmers indicating increased odds of risks.

**Table 4:** Determinants of the effects of household vectors among children.

Variable		Effect of insects					
		No	Yes	COR (95% CI)	value of *p*	AOR (95% CI)	value of *p*
Educational status of caregivers	No formal education	229	140	0.65(0.42, 1.02)	0.053	0.64(0.41,0.99)	0.047*
	Primary education	175	97	0.72(0.46,1.01)	0.15	0.70(0.45,1.12)	0.14
	More than secondary education	93	37	1			
Occupational status	Housewife	96	48	2.15(1.24,3.74)	0.006	2.21(1.23,3.70)	0.008*
	Student	82	39	2.26(124,4.02)	0.005	2.24(1.26.3.40)	0.006
	Private	42	27	1.67(0.88,3.22)	0.12	1.58(0.82, 3.05)	0.17
	Farmer	237	117	2.2(1.34, 3.53)	0.002	2.20(1.35,3.56)	0.002
	Gov. worker	40	43	1			
Accessibility of information		32	98				
		465	176	0.12(0.08,0.19)	0.00	0.12(0.08, 0.20)	0.00
Knows ways about the effects of household insects		4	85				
		493	189	0.02(0.01, 0.5)	0.00	0.014(0.01,0.04)	0.00
Handwashing facilities		376	218	1			
		121	56	0.80(0.56,1.14)	0.22	0.72(0.49–1.1)	0.105
*Per capita* water consumption, letter	Greater than 20	164	71	1			
	Less 20	333	203	1.41(1.01, 1.97)	0.04	1.45(1.02,2.07)	0.039*
Housing conditions, cleanness, by observation	Clean	9	274	1			
	Poor	488	0	1.4(0.99,1.92)	0.054		
Management of domestic waste	Liquid waste	132	76	1			
		365	198	0.99(0.95,1.04)	0.73		
	Solid waste	88	47				
		409	227	1.02(0.95, 1.1)	0.084		
Home shared with domestic animals		152	76	0.98(0.95, 1.03)	0.513		
		345	198	1			

COR, crude odds ratio; AOR, adjusted odds ratio; *significant at *p* value < 0.05, CI, confidence interval.

Additionally, the effects of household vectors were lower by 88% (AOR 0.12, 95% CI 0.08, 0.20) in households to affect those households with limited access to information about the effects of identified household vectors compared to households with information access. Additionally, the effect of household vectors was 98.6% lower among those with limited awareness compared to their counterparts or households with some awareness (AOR 0.014; 95% CI 0.01–0.04) were lesser among mothers who had limited awareness. Children from families that consumed less than 20 liters of water per person were 1.45 times more likely to be at risk compared to children from families that consumed more (AOR 1.45, 95% CI 1.02, 2.07).

### Summary of correspondence analysis

**Figure 2:**
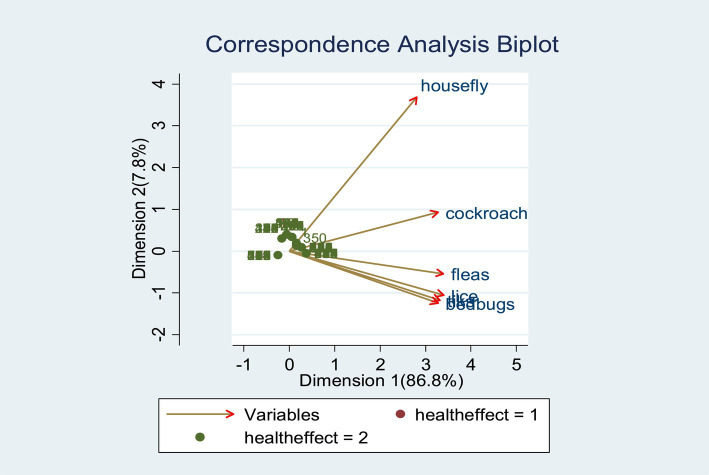
Biplot of correspondence analysis.

The correspondence analysis biplot of 771 observations and 6 variables (household vectors) indicated the effects of household vectors among the children was 86.8% explained variance by dimension 1 followed by dimension 2 (7.8%) with a total explained variance of 94.6% (Figure 2). The statistically significant level of effects/association of vectors with child health also varies (Table 5).

**Table 5:** Summary of correspondence analysis.

Correspondence analysis	
Biplot of 771 observations and 6 household vectors	
Explained variance by dimension 1	86.8
Explained variance by dimension 2	7.8
Total explained variance	94.6
Significant level of effects/association of vectors with child health effects (*p* = 0.05)	
Housefly	0.002
Cockroaches	0.042
Fleas	0.105
Lice	0.230
Bedbugs	0.083
Ticks	0.012

## Discussion

This study revealed that the effect of household vectors among children was 35.5%, which is similar to the study from a previous study that revealed household vectors are a potential vector for a diverse range of pathogenic agents (2). The household vectors that transmit disease can easily contaminate food by leaving droppings that may contain bacteria that can cause food poisoning and other pathogenic organisms (17–19) because their nocturnal and filthy habits make them ideal carriers of various pathogenic microorganisms (2, 9).

According to studies, 80% of people are allergic to dust mites, and 30% of people are allergic to cockroaches. Allergies caused by these vectors can cause several health problems, but they can be easily avoided if they are away from home (20, 21). Both vectors secrete allergens that can cause several health issues, including nasal congestion, asthma, and dermatitis. Vectors are a common cause of infections at home, particularly in Japan, they are the most frequently detected insect among all detectable insects, present at a frequency of about 90% in dust samples (22). The most important risk factor for insect allergies of any kind in people is exposure, either by sting, bite, respiratory, or dietary encounter (23). Like rats, cockroaches, ticks and fleas are known to carry salmonella, *E. coli* bacteria, and Lyme disease. Depending on the pest, the consequences of these bites and stings could range from small sores to very costly hospitalizations (24, 25).

Additionally, household vectors, such as flies, and cockroaches, can have a significant impact on child health. These vectors are capable of transmitting various diseases and infections, posing a threat to the well-being of children within the household. The association between household vectors and child health can be seen in several ways: allergies and respiratory problems: Cockroaches and dust mites are common household vectors that can trigger allergies and asthma in children. Exposure to cockroach allergens has been linked to an increased risk of respiratory symptoms, including wheezing and coughing, in children with asthma. Household vectors can indirectly contribute to malnutrition by contaminating food sources with bacteria or parasites. Flies, for instance, can transfer pathogens to uncovered food, leading to foodborne illnesses that can result in malnutrition if left untreated (5, 26, 27).

This study identified the educational status of caregivers who were not formally educated and were more at risk for the effects of common household vectors on child health. Besides the previous study reveals that respondents did not have adequate knowledge of insects which appears to weaken their knowledge of the association between insect control, prevention and other interventions (28). Awareness of those interventions on insects is not enough people should also be educated on insects and the mechanism of the control strategies.

This study reaffirms those households that have no access to information and do not know the ways about the effects of household insects are more at risk for the effects of household vectors. Informed households can employ various methods for vector control, such as using insecticides or implementing integrated pest management techniques. Individuals who lack knowledge may resort to ineffective or harmful methods that can intensify the problem or pose health risks to them. Preventive practices of household vectors were significantly correlated with the attitude and knowledge of individual households (29). Evidence shows household vectors are endemic both in developing and developed countries and affect persons of all ages and socioeconomic backgrounds particularly infestation occurs most commonly in children (30). The high prevalence was associated with the education status of the female head and close supervision of households by health extension workers (31).

This study shows the occupational status of the household being housewife, student and farmer are more at risk for the effects of household insects are common in resource-poor communities in South America and sub-Saharan Africa with prevalence in the general population of up to 60% (32). Besides effects of households on child health are common in vulnerable groups and economically disadvantaged communities in which such groups may not have enough income for the intervention of household insect effects, especially among lower socioeconomic groups (33), which may be mediated by socioeconomic status and other household environmental factors.

Moreover, this study showed that the unavailability of handwashing facilities and consumption of less than 20 liter *per capita* water is the likely risk factor for the effects of household vectors on child health. Unavailability of adequate water for households may not be sufficient for family domestic water uses such as drinking, and keeping personal hygiene including washing child clothes to prevent infestation of vectors that causes different health outcomes.

The limitation of the study is the qualitative research methods are not used to provide a more comprehensive understanding of the cause effects of the household vectors on children’s health.

## Conclusion

There was a high burden of household vectors on child health in resource-limited areas of southwestern Ethiopia. The burden was significantly affected by factors such as the educational and occupational status of caregivers, accessibility of information, awareness of household on household vectors, and *per capita* water consumption of households. Hence: the health sector should focus on strengthening health communication activities to create awareness of household vectors. Improving environmental strategies would contribute to the reduction of the effects of vectors in resource-limited areas; future research should be done on the mechanism of effects of household vectors on children.

## Data availability statement

The raw data supporting the conclusions of this article will be made available by the authors, without undue reservation.

## Ethics statement

The studies involving humans were approved by the Mizan-Tepi University Institutional Research Review Board. The studies were conducted in accordance with the local legislation and institutional requirements. The participants provided their written informed consent to participate in this study.

## Author contributions

BA: Conceptualization, Data curation, Formal analysis, Funding acquisition, Investigation, Methodology, Project administration, Resources, Software, Supervision, Validation, Visualization, Writing – original draft, Writing – review & editing. BM: Conceptualization, Data curation, Formal analysis, Investigation, Methodology, Project administration, Resources, Software, Supervision, Validation, Visualization, Writing – original draft, Writing – review & editing. AEA: Conceptualization, Data curation, Formal analysis, Investigation, Methodology, Resources, Software, Supervision, Validation, Writing – review & editing. AYA: Conceptualization, Data curation, Funding acquisition, Investigation, Methodology, Project administration, Resources, Software, Supervision, Visualization, Writing – review & editing, Writing – original draft.
